# Beyond weight loss: predictors of treatment satisfaction and patient-reported outcomes in incretin-based therapies. A cross-sectional study

**DOI:** 10.3389/fendo.2026.1864209

**Published:** 2026-06-17

**Authors:** Ali Kapan, Othmar Moser, Richard Felsinger, Thomas Waldhoer

**Affiliations:** 1 Center for Public Health, Department of Social and Preventive Medicine, Medical University of Vienna, Vienna, Austria; 2 Exercise Physiology, Training and Training Therapy Research Group, Institute of Human Movement Science, Sport and Health, University of Graz, Graz, Austria; 3 Division of Endocrinology and Diabetology, Department of Internal Medicine, Medical University of Graz, Graz, Austria; 4 Center for Public Health, Department of Epidemiology, Medical University of Vienna, Vienna, Austria

**Keywords:** GLP-1 receptor agonists, incretin-based therapy, obesity, patient-reported outcomes, tirzepatide, treatment satisfaction

## Abstract

**Background:**

Incretin-based therapies, including glucagon-like peptide-1 receptor agonists and dual glucose-dependent insulinotropic polypeptide/glucagon-like peptide-1 receptor agonists, have demonstrated substantial efficacy in weight management. However, real-world treatment experience, tolerability, and treatment continuation remain important challenges, and the determinants of patient satisfaction are not fully understood.

**Methods:**

A cross-sectional online survey was conducted between January and February 2025 across international incretin therapy communities. The study included 411 adults with obesity (BMI ≥30 kg/m²) or overweight with comorbidities (BMI 27–29.9 kg/m²) receiving semaglutide or tirzepatide for ≥3 months. Treatment satisfaction was assessed using the validated Treatment-Related Impact Measure–Weight (TRIM-Weight). Patient-reported changes in appetite regulation, mood, physical activity, sexual health, self-image, and social experiences were evaluated using 5-point Likert scales.

**Results:**

Participants had a median age of 39 years (IQR 33–47), 71.8% were female, and the median treatment duration was 10 months (IQR 7–13), with 34.3% reporting treatment for ≥12 months. In univariate analyses, higher treatment satisfaction was associated with improvements in appetite regulation, mood, physical activity, sexual satisfaction, self-image, and positive social feedback, as well as fewer limitations due to side effects (all p < 0.05). In fully adjusted multivariable models, fewer limitations due to side effects (β = 1.41; 95% CI 0.25–2.88), increased satiety (β = 0.68; 95% CI 0.10–1.29), improved mood (β = 0.69; 95% CI 0.19–1.28), and higher physical activity (β = 0.58; 95% CI 0.10–1.17) remained independently associated with higher TRIM-Weight scores. Greater BMI reduction was associated with higher treatment satisfaction in the individually adjusted model, but this association was attenuated and no longer statistically significant in the fully adjusted model.

**Conclusions:**

Treatment satisfaction was more strongly associated with tolerability, satiety, mood, and physical activity than with BMI change or medication type. These findings support incorporating patient-reported outcomes when evaluating incretin-based therapies in real-world obesity care.

## Introduction

The introduction of glucagon-like peptide-1 receptor agonists (GLP-1 RAs), such as semaglutide, and the dual glucose-dependent insulinotropic polypeptide and GLP-1 receptor agonist (GIP/GLP-1 RAs) tirzepatide has marked a paradigm shift in the therapeutic management of type 2 diabetes, obesity, overweight, and other cardiometabolic diseases (1, 2). Semaglutide is approved for glycaemic control in type 2 diabetes and for chronic weight management, whilst tirzepatide is approved for type 2 diabetes and, in some jurisdictions, for chronic weight management (3). In the STEP trials (3), semaglutide (2.4 mg once weekly) achieved average weight reductions of 14-16% in individuals without diabetes and approximately 10% in individuals with type 2 diabetes, accompanied by clinically meaningful reductions in HbA1c of 1.5–1.8 percentage points in the SUSTAIN programme (4). Substantial weight loss of a greater magnitude has historically been achieved with bariatric surgery, with 5-year total weight loss of 22.5% after sleeve gastrectomy and 26.0% after Roux-en-Y gastric bypass (5).

Despite these substantial clinical benefits, real-world persistence with incretin-based therapies remains variable. Gleason et al. (6) found that amongst individuals with obesity without type 2 diabetes, only 32.3% remained on GLP-1 RA therapy after one year, with an adherence rate of 27.2%. Discontinuation rates have also been reported to be higher in individuals without type 2 diabetes than in those with type 2 diabetes (7). Reasons for discontinuation are heterogeneous and may include gastrointestinal side effects, cost or insurance-related access barriers, and patients’ perceptions of whether continued treatment is necessary after achieving weight-loss goal (8, 9). These factors indicate that persistence is influenced not only by medication efficacy, but also by tolerability, access, and patient experience.

Importantly, treatment discontinuation is frequently associated with weight regain and a loss of previously achieved metabolic and psychosocial benefits. For example, in the STEP-4 study (10), improvements in quality of life were sustained over two years with continued semaglutide treatment, whereas discontinuation was often associated with weight regain and deterioration in QoL. This discrepancy between high efficacy in clinical trials and limited persistence in real-world settings highlights a critical gap in long-term treatment success. Patient satisfaction, which is influenced by psychosocial outcomes and treatment experience, may contribute to treatment continuation and persistence (11). In this context, understanding how patient-reported outcomes (PROs) contribute to satisfaction and treatment continuation becomes essential. In addition to the well-documented reductions in body weight and HbA1c levels, patient satisfaction has increasingly been recognised as an important determinant of treatment success (12, 13). Dimensions such as treatment satisfaction, quality of life (QoL) and psychosocial outcomes are now recognised as critical factors in achieving long-term therapeutic success (14). A randomised placebo-controlled trial has shown that GLP-1 RAs have profound effects on eating behaviour, including a reduction in hunger and food cravings and an earlier onset of satiety (15). These behavioural changes are associated with significant improvements in psychosocial parameters, including a statistically significant increase in mental health scores as measured by the SF-36 (16). Similarly, the SURPASS-6 study reported that tirzepatide led to significant improvements over insulin lispro in all domains of the 36-Item Short Form Health Survey Version 2 (SF-36v2), particularly in general health, vitality and mental health, as well as benefits in patient-reported outcomes (PROs) such as the Ability to Perform Physical Activities of Daily Living (APPADL), Impact of Weight on Self-Perceptions (IW-SP) and the EuroQol 5-Dimensions 5-Levels Questionnaire (EQ-5D-5L) (17). However, most prior studies have used standardised instruments in controlled trial settings, with limited attention to the broader psychosocial experiences of patients in real-world care. It remains unclear which specific patient-reported domains, including appetite regulation, mood, self-image, and social experiences, are most strongly associated with treatment satisfaction. This study addresses these gaps by examining psychosocial and treatment-related experiences in a real-world, social media-recruited sample and by identifying which PRO domains are most strongly associated with TRIM-Weight treatment satisfaction.

## Methods

The present study is a secondary analysis utilizing a cross-sectional design. Data were collected via a structured questionnaire disseminated between January 13 and February 28, 2025, across various social media platforms, including Facebook^®^, Instagram^®^, X^®^, and WhatsApp^®^. Recruitment specifically targeted online communities and support groups for individuals using GLP-1 or dual GIP/GLP-1 receptor agonists (RAs). Collectively, these targeted groups encompassed an estimated potential reach of over 400,000 members across Europe and North America, resulting in an international sample without restriction to a specific country. The study was approved by a local ethics committee. Participants were required to provide informed consent prior to participation by selecting the “Agree” option, thereby confirming that they had read and understood the participant information sheet and met the stated eligibility criteria. It was made clear that participation was voluntary, and that respondents could discontinue the survey at any point without any consequences. Furthermore, participants were informed that all collected data would be used solely for scientific research purposes. The administration of the questionnaire was conducted online via the SoSci Survey platform (https://www.soscisurvey.de). To ensure data integrity and prevent automated responses (bots) or multiple submissions, a CAPTCHA verification step was implemented. The development and deployment of the survey adhered to the Checklist for Reporting Results of Internet E-Surveys guidelines (18) to ensure quality, transparency, and data protection throughout the online survey process. The online survey required approximately 20 minutes to complete. No financial compensation or incentives were provided for participation.

### Eligibility criteria

Participants were recruited through online platforms with clearly defined inclusion and exclusion criteria. Eligibility requirements were verified through automated filter logic within the survey platform, and only complete responses were included in the final dataset. The study population comprised adults (≥18 years) meeting specific clinical criteria for incretin therapy initiation. Participants were eligible if they self-reported a history of obesity, defined as an initial BMI ≥30 kg/m², or overweight with weight-related comorbidities, defined as BMI 27–29.9 kg/m² with at least one relevant comorbidity. Active treatment with GLP-1 receptor agonists or dual GIP/GLP-1 receptor agonists was mandatory, with a minimum treatment duration of three consecutive months prior to survey completion. For the purpose of this study, medication exposure was assessed using the brand names of the incretin-based therapies most widely available and recognised across Europe and North America at the time of the survey design (i.e., early 2025), specifically Ozempic^®^ and Wegovy^®^ (semaglutide) as well as Mounjaro^®^ (tirzepatide). Other GLP-1 receptor agonists (e.g., liraglutide) or more recently introduced alternative formulations of tirzepatide for obesity (e.g., Zepbound^®^) were not explicitly captured in the survey instrument. Additional requirements included English language proficiency for questionnaire completion and capacity to provide informed consent.

### Questionnaire content

To capture both clinical and contextual aspects of incretin-based therapies, the questionnaire collected detailed information on socio-demographic and health-related characteristics. These included participants’ age, sex, income, education and employment status, as well as self-reported dietary habits and frequency of moderate-to-vigorous physical activity since starting treatment. Clinical data included self-reported height and weight at baseline and at the time of the survey. Participants provided information on the type, dosage and duration of their current treatment with incretin-based medications, as well as the medical specialty of the prescribing physician. The presence of common weight-related comorbidities (particularly type 2 diabetes, hypertension, dyslipidaemia, sleep apnoea, non-alcoholic fatty liver disease, osteoarthritis and stroke) was also recorded.

In addition, to assess perceived changes since the initiation of incretin-based therapy, a structured questionnaire was developed that covered multiple domains. The selection of these domains was informed by prior literature on incretin-based therapies and obesity-related patient-reported outcomes, demonstrating that GLP-1 receptor agonists influence appetite regulation, including hunger, food craving and satiety (10). Furthermore, established patient-reported outcome frameworks in obesity research, such as the BODY-Q, highlight the importance of assessing domains including eating behaviour, physical and psychological well-being, body image, and social functioning (19). These included changes in appetite regulation (appetite, food craving, satiety, portion size, fluid intake), psychological and physical well-being (mood, energy, sleep, libido, satisfaction with sexual life), as well as self-image, social reactions, and aesthetic perception (e.g., perceived changes in facial appearance). Appearance-related perceptions were included as part of broader body image constructs, which have been shown to be key determinants of quality of life and psychosocial outcomes in individuals with obesity and following weight loss (20). All questions were phrased in a comparative format (e.g., “Compared to before starting incretin therapy…”) and answered using five-point ordinal Likert-type scales. The response options ranged from 1 = very negative change (e.g., “strongly worsened,” “strongly decreased,” “very dissatisfied”) to 5 = very positive change (e.g., “strongly improved,” “strongly increased,” “very satisfied”). Higher scores consistently indicated more favourable changes or perceptions, unless otherwise specified.

### Treatment-related impact measure – weight

To assess the perceived impact of pharmacological weight management, the study employed the Treatment-Related Impact Measure – Weight (TRIM-Weight), a validated PRO instrument developed specifically for individuals receiving weight-related therapy. The TRIM-Weight captures subjective treatment experiences across five domains: perceived control over weight management, impact on daily life, treatment burden, experience of side effects, and psychological well-being. These domains jointly reflect how the therapy integrates into everyday life, the extent to which it is perceived as burdensome or disruptive, and its influence on both physical and emotional well-being. Item responses were coded and scored according to the TRIM-Weight scoring manual distributed by the Mapi Research Trust on behalf of the instrument developer (21), with reverse coding applied where required. Domain scores were calculated and transformed to a standardised 0–100 scale as specified in the scoring guidelines, with higher scores indicating more favourable outcomes. Use of the TRIM-Weight instrument in this study was authorised by Mapi Research Trust via the ePROVIDE™ platform (User License Agreement on file). The instrument was applied in accordance with the licensing terms, without modifications or translation (21).

### Sample size

As this was an exploratory study, no formal *a priori* power analysis was performed. We employed convenience sampling through online platforms to maximise participant recruitment. The resulting sample size (n = 411) was considered adequate to identify relevant associations, consistent with feasibility-driven, hypothesis-generating real-world studies.

### Statistics

Descriptive statistics were first computed to summarise the sample characteristics. Depending on data distribution, continuous variables were presented as means with standard deviations (SDs) or medians with interquartile ranges (IQRs). Normality of continuous variables was assessed using histograms and the Shapiro–Wilk test. Categorical variables were reported as absolute and relative frequencies (n, %).

To examine associations between patient-reported experiences and treatment satisfaction, the TRIM-Weight Total Score (range 0–100) served as the dependent variable in all models. In a first analytical step, separate univariate one-way ANOVAs were conducted for each patient-reported change variable. These included perceived changes in appetite and eating behaviours (food cravings, appetite, portion size, and timing of satiety), physical and psychological well-being (mood, energy levels, sleep quality, self-image, and physical activity), sexual health (libido and sexual satisfaction), social experiences (feedback from others, changes in social interaction, and social media sharing), and treatment-related perceptions (daily impact of side effects, facial appearance, enjoyment of meals, treatment continuation intentions, perceived cost barriers, and long-term safety concerns). For each variable, mean TRIM-Weight scores were compared across response categories. Where the overall ANOVA was significant (p < 0.05), Bonferroni-corrected *post hoc* comparisons were performed. Variables that were not significantly associated with the TRIM-Weight total score in the initial ANOVA analyses were not considered for further modelling. Given the large number of significant variables across related domains, not all were included simultaneously. Instead, conceptually overlapping variables were reviewed, and a subset of clinically relevant and representative predictors was selected to avoid redundancy and reduce the risk of overfitting.

In the second step, selected candidate variables were entered into separate multivariable linear regression models (Model 1) to examine their associations with the TRIM-Weight total score after adjustment for covariates. Model 2 was then used to determine which variables were independently associated with TRIM-Weight scores when all candidate predictors were considered simultaneously. Semaglutide was used as the reference group in the regression analyses because it represented the larger subgroup. All models were adjusted for age, sex, baseline BMI, treatment duration, comorbidities and medication dose to account for treatment intensity. The regression results are presented as β coefficients with 95% confidence intervals (CIs), and a two-sided p-value of less than 0.05 was considered to be statistically significant. To assess potential multicollinearity amongst predictors in Model 2, the variance inflation factor (VIF) was calculated; all values were below 5, indicating no evidence of problematic multicollinearity. There were no missing data. All analyses were performed using SPSS version 28.0 (IBM Corp., Armonk, NY).

## Results

A total of 676 individuals initiated the survey, of whom 411 (60.8%) completed all required sections and were included in the final analysis. The remaining 265 participants (39.2%) were excluded due to incomplete responses, primarily resulting from early termination during initial stages of the survey, such as eligibility screening or sociodemographic and anthropometric questions. Consequently, key outcome measures, including TRIM-Weight scores, treatment experiences, and side effects, were not available for these individuals. The excluded participants were predominantly female (72%), with a median age of 37 years (IQR: 28–50) and a median baseline BMI of 34.4 kg/m² (IQR: 31.4–39.6). The final analytic sample was geographically diverse, with the largest proportions of participants residing in the United States (n = 111, 27%), the United Kingdom (n = 96, 23%), and Germany (n = 80, 20%). Additional participants were recruited from Canada (n = 36, 9%), Australia (n = 26, 6%), Spain (n = 18, 4%), Portugal (n = 6, 2%), and other European countries (n = 38, 9%).

Sex distribution was similar between groups, with women representing 71.2% of participants with type 2 diabetes and 72.1% of those without type 2 diabetes (**Table 1**). **Table 2** shows that the most frequently used medication was semaglutide 2.4 mg/week (217 participants; 52.8%), followed by semaglutide 1.0 mg/week (148; 36.0%), tirzepatide 10 mg/week (31; 7.5%), and tirzepatide 12.5 mg/week (15; 3.6%). Full cost coverage was reported exclusively by participants with type 2 diabetes (50 participants; 34.2%), whereas no cost coverage was reported by 252 participants without type 2 diabetes (95.1%). Participants without type 2 diabetes had a longer median treatment duration (11 vs. 9 months) and a greater median reduction in BMI (6.3 vs. 4.8 kg/m²). However, monthly weight loss was comparable between the two groups (1.5 kg per month). Overall, 141 participants (34.3%) had received treatment for at least 12 months. TRIM-Weight total scores were broadly comparable between participants with and without type 2 diabetes (66.1 ± 7.3 vs. 68.2 ± 7.0), with only minor differences across most subdomains. Exploratory analyses by sex showed largely comparable TRIM-Weight scores between male and female participants. Only the Weight Management domain differed significantly by sex (mean difference: 4.74 points; p = 0.012).

**Table 1:** Sociodemographic and clinical characteristics by type 2 diabetes status.

Variables	All (n = 411)	Type 2 diabetes (n = 146)	No type 2 diabetes (n = 265)
Age, years, median (IQR)	39 (33–47)	37 (31–48)	41 (36–44)
Sex, n (%)			
Female	295 (71.8)	104 (71.2)	191 (72.1)
Male	116 (28.2)	42 (28.8)	74 (27.9)
Marital status, n (%)			
Married	73 (17.8)	34 (23.3)	39 (14.7)
Divorced	73 (17.8)	30 (20.5)	43 (16.2)
Single	125 (30.4)	38 (26.0)	87 (32.8)
Widowed	1 (0.2)	0 (0.0)	1 (0.4)
In a relationship	139 (33.8)	44 (30.1)	95 (35.8)
Monthly income, n (%)			
< 500 €	14 (3.4)	5 (3.4)	9 (3.4)
500 € to < 1000 €	20 (4.9)	12 (8.2)	8 (3.0)
1000 € to < 1500 €	42 (10.2)	15 (10.3)	27 (10.2)
1500 € to < 2000 €	86 (20.9)	29 (19.9)	57 (21.5)
2000 € to < 3000 €	183 (44.5)	62 (42.5)	121 (45.7)
3000 € to < 4000 €	24 (5.8)	6 (4.1)	18 (6.8)
4000 € to < 5000 €	4 (1.0)	2 (1.4)	2 (0.8)
≥ 5000 €	12 (2.9)	2 (1.4)	10 (3.8)
Prefer not to answer	26 (6.3)	13 (8.9)	13 (4.9)
Education, n (%)			
Compulsory/lower secondary school	46 (11.2)	25 (17.1)	21 (7.9)
Vocational training/apprenticeship	100 (24.3)	36 (24.7)	64 (24.2)
General higher education entrance qualification	128 (31.1)	48 (32.9)	80 (30.2)
University degree	137 (33.3)	37 (25.3)	100 (37.7)
Employment status, n (%)			
Training/apprenticeship	22 (5.4)	14 (9.6)	8 (3.0)
University student	26 (6.3)	13 (8.9)	13 (4.9)
Employee	297 (72.3)	91 (62.3)	206 (77.7)
Self-employed	60 (14.6)	26 (17.8)	34 (12.8)
Prefer not to answer	6 (1.5)	2 (1.4)	4 (1.5)
Comorbidities, n (%)			
Hypertension	140 (34.1)	66 (45.2)	74 (27.9)
Sleep apnoea	39 (9.5)	7 (4.8)	32 (12.1)
Dyslipidaemia	141 (34.3)	56 (38.4)	85 (32.1)
Non-alcoholic fatty liver disease	33 (8.0)	22 (15.1)	11 (4.2)
Stroke	28 (6.8)	8 (5.5)	20 (7.5)
Knee osteoarthritis	80 (19.5)	35 (24.0)	45 (17.0)

Values are presented as n (%) for categorical variables and as median and interquartile range (IQR) for continuous variables.

**Table 2:** Treatment characteristics, anthropometric outcomes, adverse events, and patient-reported outcomes by type 2 diabetes status.

Variables	All (n = 411)	Type 2 diabetes (n = 146)	No type 2 diabetes (n = 265)
Incretin therapy, n (%)			
Semaglutide 2.4 mg/week	217 (52.8)	69 (47.3)	148 (55.8)
Semaglutide 1.0 mg/week	148 (36.0)	63 (43.2)	85 (32.1)
Tirzepatide 10 mg/week	31 (7.5)	11 (7.5)	20 (7.5)
Tirzepatide 12.5 mg/week	15 (3.6)	3 (2.1)	12 (4.5)
Cost coverage, n (%)			
Full coverage	50 (12.2)	50 (34.2)	0 (0.0)
Partial coverage	109 (26.5)	96 (65.8)	13 (4.9)
No coverage	252 (61.3)	0 (0.0)	252 (95.1)
Duration of therapy, months, median (IQR)	10 (7–13)	9 (6–12)	11 (8–14)
Duration of therapy in 3-month categories, n (%)			
3 to <6 months	78 (19.0)	42 (28.8)	36 (13.6)
6 to <9 months	97 (23.6)	31 (21.2)	66 (24.9)
9 to <12 months	95 (23.1)	37 (25.3)	58 (21.9)
≥12 months	141 (34.3)	36 (24.7)	105 (39.6)
Anthropometric measurements, median (IQR)			
Height, cm	169.0 (163.0–177.0)	169.0 (164.0–174.0)	171.0 (163.0–177.0)
Weight, kg	105.5 (92.5–120.0)	100.5 (91.7–118.3)	107.9 (92.9–120.8)
BMI at baseline, kg/m²	36.4 (32.6–41.4)	35.0 (31.6–40.7)	37.0 (33.3–42.1)
BMI at follow-up, kg/m²	30.9 (27.6–35.2)	30.2 (27.4–34.7)	31.0 (27.8–35.4)
Delta BMI, kg/m²	6.0 (4.0–7.4)	4.8 (3.3–6.8)	6.3 (4.5–7.8)
Weight loss, kg/month	1.5 (1.3–1.9)	1.5 (1.3–1.8)	1.5 (1.3–1.9)
Adverse events during incretin therapy, n (%)			
Nausea	147 (35.8)	66 (45.2)	81 (30.6)
Constipation	112 (27.3)	30 (20.5)	82 (30.9)
Diarrhoea	109 (26.5)	33 (22.6)	76 (28.7)
Vomiting	107 (26.0)	37 (25.3)	70 (26.4)
Eructation	89 (21.7)	31 (21.2)	58 (21.9)
Depressed mood	80 (19.5)	23 (15.8)	57 (21.5)
Fatigue	64 (15.6)	26 (17.8)	38 (14.3)
Nasopharyngitis	63 (15.3)	32 (21.9)	31 (11.7)
Injection-site haematoma	51 (12.4)	21 (14.4)	30 (11.3)
Dizziness	49 (11.9)	19 (13.0)	30 (11.3)
Abdominal pain	48 (11.7)	20 (13.7)	28 (10.6)
TRIM-Weight scores, mean ± SD			
TRIM-Weight Total Score	68.2 ± 7.1	66.1 ± 7.3	68.2 ± 7.0
Daily Life	82.7 ± 9.3	81.2 ± 8.5	83.3 ± 9.8
Weight Management	67.2 ± 17.2	65.7 ± 17.7	67.5 ± 16.9
Treatment Burden	59.8 ± 18.5	60.5 ± 17.3	55.9 ± 19.1
Experience of Side Effects	44.8 ± 13.3	45.7 ± 14.0	44.9 ± 12.9
Psychological Health	81.7 ± 16.8	80.2 ± 16.4	82.0 ± 17.0

Values are presented as n (%) for categorical variables and as median and interquartile range (IQR) for continuous variables.

**Figure 1:**
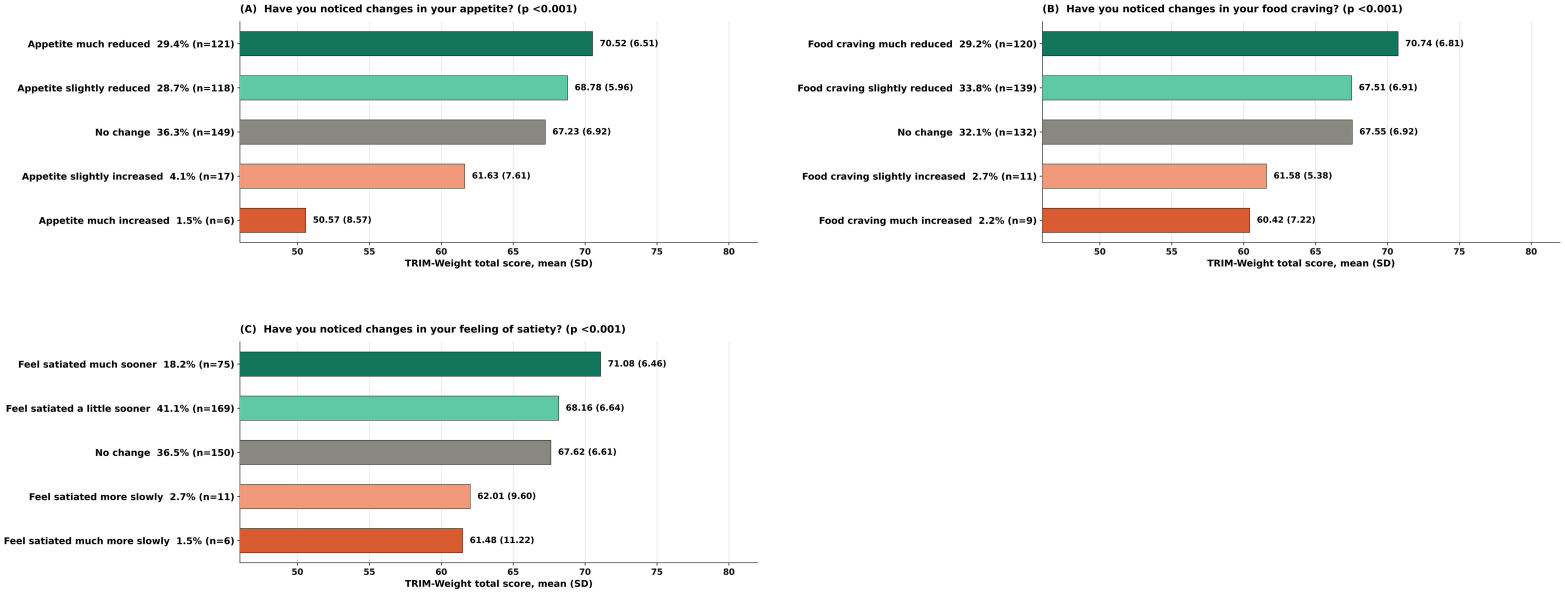
Patient-reported changes in appetite, food craving, and satiety and treatment satisfaction. Data are presented as n for each response category and mean (standard deviation, SD) for TRIM-Weight total scores. Group differences in TRIM-Weight total scores across response categories were assessed using one-way analysis of variance (ANOVA). Where the overall ANOVA was statistically significant (p < 0.05), Bonferroni-corrected *post hoc* comparisons were performed to identify pairwise group differences whilst controlling for multiple testing. **(A)** Self-reported changes in appetite since initiation of incretin-based therapy. **(B)** Self-reported changes in food craving since initiation of incretin-based therapy. **(C)** Self-reported changes in satiety since initiation of incretin-based therapy.

**Figure 1** shows that participants who reported a much-reduced appetite had the highest satisfaction scores (mean = 70.52; SD = 6.51), followed by those with a slightly reduced appetite (mean = 68.78; SD = 5.96). Those with a much-increased appetite showed the lowest scores (50.57; SD = 8.57; p < 0.001). Food craving reduction followed a similar pattern, with much reduced cravings achieving higher satisfaction (70.74; SD = 6.81) versus increased cravings (60.42; SD = 7.22; p < 0.001). Earlier satiety onset was associated with higher TRIM-Weight scores (71.08; SD = 6.46) compared to delayed satiety (61.48; SD = 11.22; p < 0.001).

**Figure 2:**
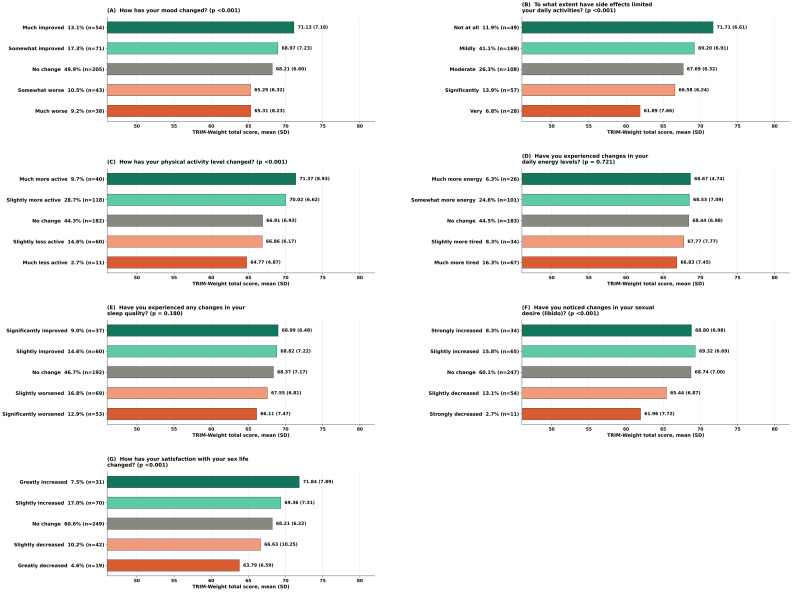
Patient-reported changes in physical and emotional functioning and treatment satisfaction. Data are presented as n for each response category and mean (standard deviation, SD) for TRIM-Weight total scores. Group differences in TRIM-Weight total scores across response categories were assessed using one-way analysis of variance (ANOVA). Where the overall ANOVA was statistically significant (p < 0.05), Bonferroni-corrected *post hoc* comparisons were performed to identify pairwise group differences whilst controlling for multiple testing. **(A)** Self-reported changes in mood since initiation of incretin-based therapy. **(B)** Self-reported impact of side effects on daily activities. **(C)** Self-reported changes in physical activity levels since initiation of therapy. **(D)** Self-reported changes in daily energy levels since initiation of therapy. **(E)** Self-reported changes in sleep quality since initiation of therapy. **(F)** Self-reported changes in sexual desire (libido) since initiation of therapy. **(G)** Self-reported changes in satisfaction with sex life since initiation of therapy.

**Figure 2** shows that improvements in mood, physical activity and sexual health, as well as fewer limitations related to side effects, were associated with higher TRIM-Weight scores (all p < 0.001). Participants who reported much-improved mood or physical activity had the highest satisfaction scores. In contrast, severe side-effect-related limitations and declines in libido or sexual satisfaction were associated with lower scores.

**Figure 3:**
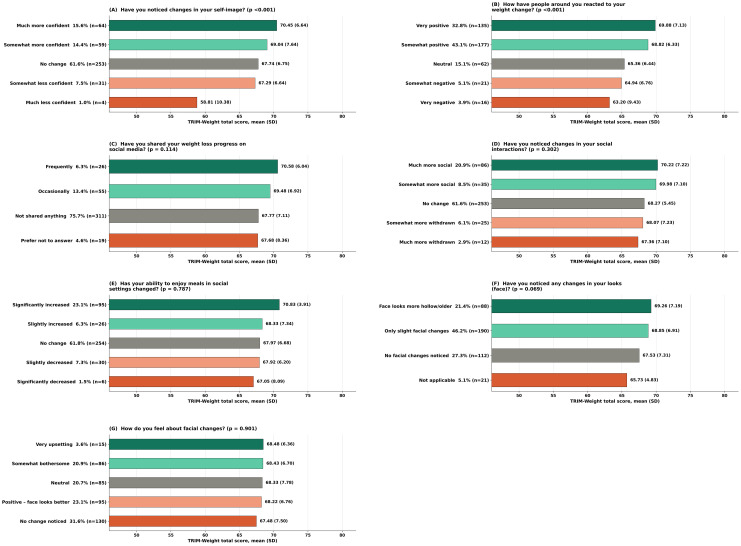
Patient-reported psychosocial changes and treatment satisfaction. Data are presented as n for each response category and mean (standard deviation, SD) for TRIM-Weight total scores. Group differences in TRIM-Weight total scores across response categories were assessed using one-way analysis of variance (ANOVA). Where the overall ANOVA was statistically significant (p < 0.05), Bonferroni-corrected *post hoc* comparisons were performed to identify pairwise group differences whilst controlling for multiple testing. **(A)** Self-reported changes in self-image since initiation of incretin-based therapy. **(B)** Perceived reactions of others to weight change. **(C)** Sharing of weight loss progress on social media. **(D)** Self-reported changes in social interactions. **(E)** Self-reported changes in enjoyment of meals in social settings. **(F)** Self-reported changes in facial appearance. **(G)** Emotional response to facial changes.

**Figure 3** shows that improved self-image was a strong predictor of satisfaction (p < 0.001). Participants who felt much more confident had the highest TRIM-Weight scores (mean = 70.45; SD = 6.64), whereas those who felt much less confident had markedly lower scores (mean = 58.81; SD = 10.38). Similarly, participants who received very positive feedback from others about their weight loss reported higher satisfaction (mean = 69.88; SD = 7.13) than those who received very negative feedback (mean = 63.20; SD = 9.43; p < 0.001). **Table 3** shows the results of two multivariable linear regression models assessing factors associated with the total TRIM-Weight score. In Model 1, all variables were significantly associated with higher TRIM-Weight scores. In the fully adjusted model, which included all predictors simultaneously, fewer limitations due to side effects (β = 1.41; 95% CI, 0.25–2.88; p = 0.01), increased satiety (β = 0.68; 95% CI, 0.10–1.29; p = 0.05), improved mood (β = 0.69; 95% CI, 0.19–1.28; p = 0.05), and higher physical activity (β = 0.58; 95% CI, 0.10–1.17; p = 0.05) remained independently associated with higher TRIM-Weight scores. However, BMI reduction was attenuated and was no longer statistically significant after adjusting for patient-reported treatment experiences and covariates simultaneously.

**Table 3:** Associations between patient characteristics, perceived changes, and TRIM-weight total score.

Parameter	TRIM Weight total score			
	Multivariable model 1	R²	Multivariable model 2	R²
	β (95% CI)		β (95% CI)	0.17
Semaglutide	Reference		Reference	
Tirzepatide	1.36 (0.11 – 2.25)*	0.07	1.29 (-0.35 – 2.13)	
Delta BMI (kg/m²)	0.60 (0.02 – 1.19)*	0.05	0.46 (-0.22 – 1.16)	
Limitation due to side effects	0.76 (0.21 – 1.51)**	0.09	1.41 (0.25 – 2.88)**	
Changes in appetite	0.63 (0.11 – 1.48)**	0.06	0.47 (-0.27 – 1.25)	
Changes in craving	0.64 (0.09 – 1.41)*	0.06	0.41 (-0.20 – 1.03)	
Changes of satiety	0.87 (0.13 – 1.62)**	0.10	0.68 (0.10 – 1.29)*	
Changes in mood	0.64 (0.12 – 1.19)*	0.07	0.69 (0.19 – 1.28)*	
Changes in physical activity	0.76 (0.13 – 1.49)**	0.09	0.58 (0.10 – 1.17)*	
Changes in self image	0.54 (0.10 – 1.11)*	0.04	0.22 (-0.26 – 1.03)	
Changes in satisfaction with sex life	0.92 (0.19 – 1.60)**	0.11	0.45 (-0.31 – 1.38)	

The dependent variable in all models was the total score on the TRIM-Weight scale. Model 1 comprised separate multivariable linear regression analyses for each independent variable, each adjusted for baseline BMI, age, sex, treatment duration (weeks), comorbidities, and medication dosage. Model 2 included all listed variables simultaneously and was adjusted for the same covariates. BMI reduction was calculated as baseline BMI minus follow-up BMI; therefore, higher values indicate greater BMI reduction. Semaglutide was used as the reference category. *p < 0.05, **p < 0.01.

## Discussion

This cross-sectional study aimed to identify factors associated with treatment satisfaction amongst individuals using incretin-based therapies for weight management. Overall, treatment satisfaction was most closely associated with patient-reported treatment experience, particularly fewer side effect-related limitations, increased satiety, improved mood, and higher physical activity. BMI reduction was associated with higher TRIM-Weight scores in the adjusted single-predictor model, but this association was attenuated after full adjustment, suggesting that satisfaction was more strongly related to how patients experienced treatment than to BMI reduction alone.

An initial examination of the TRIM-Weight data (**Table 2**) provides a comprehensive overview of the general patient experience. Overall, the cohort reported a mean TRIM-Weight total score of 68.2 ± 7.1, with broadly comparable scores between participants with and without type 2 diabetes. Notably, participants scored highest in the “Daily Life” (82.7 ± 9.3) and “Psychological Health” (81.7 ± 16.8) sub-domains, suggesting that incretin therapies were perceived as relatively compatible with daily routines and were accompanied by favourable psychological ratings in this sample. The high psychosocial and daily functioning scores align with recent patient-reported outcome analyses from the STEP and SURMOUNT trial programs, which reported improvements in physical functioning and weight-related quality of life during incretin-based treatment (22, 23). In contrast, the lowest-scoring subdomain across the entire cohort was ‘Experience of Side Effects’ (44.8 ± 13.3). This lower score in the side-effect domain is consistent with the significant prevalence of gastrointestinal adverse events observed in our sample, including nausea (35.8%) and constipation (27.3%). These frequencies closely align with recent real-world observational data, suggesting that gastrointestinal symptoms remain an important treatment burden and may negatively affect patient experience outside of strictly controlled trial environments (24).

Building on this general overview, further analyses examined the associations between specific physical and psychosocial changes and overall satisfaction. In the bivariate analyses, multiple patient-reported domains including appetite, food craving, satiety, mood, physical activity, self-image, sexual satisfaction, and social feedback, as well as limitations due to side effects were significantly associated with treatment satisfaction. These associations were largely confirmed in the adjusted single-variable regression models (Model 1). In the fully adjusted multivariable model (Model 2), four variables remained independently associated with higher TRIM-Weight scores: fewer limitations due to side effects (β = 1.41), increased satiety (β = 0.68), improved mood (β = 0.69), and higher physical activity (β = 0.58). Given the cross-sectional design, these findings should be interpreted as associations rather than evidence of causal effects.

In contrast, BMI reduction (β = 0.46; 95% CI: −0.22–1.16) and medication type (tirzepatide vs. semaglutide: β = 1.29; 95% CI: −0.35–2.13) were not significantly associated with treatment satisfaction after full adjustment. These findings indicate that neither weight loss nor treatment type independently explained variability in satisfaction once patient-reported experiences were taken into account. This aligns with previous research indicating that treatment satisfaction in obesity is not solely determined by weight loss, but also associated with behavioural and psychosocial factors (25, 26). Taken together, these results suggest that treatment satisfaction may be more strongly associated with how patients experience therapy in everyday life, particularly with regard to tolerability, appetite regulation, and functional and psychological well-being, than with objective weight-related outcomes. Although tirzepatide differs mechanistically from GLP-1 receptor agonists through dual GIP and GLP-1 receptor agonism (1), clinical studies indicate that gastrointestinal adverse events remain common and dose-dependent (23, 24). In the present study, these pharmacological differences were not associated with differences in treatment satisfaction. While emerging preclinical research suggests that GIP receptor signalling may influence gastrointestinal tolerability (27), such mechanisms were not directly assessed in this study and should therefore be interpreted cautiously.

The associations observed for mood and physical activity add valuable real-world context to the evolving literature on incretin-based therapies. Evidence regarding psychiatric outcomes has historically been mixed. While recent large-scale observational analyses suggest a reduced risk of incident depression, anxiety, and suicidal ideation amongst users of GLP-1 receptor agonists (28), pharmacovigilance signals have raised concerns regarding potential mood-related adverse effects, prompting regulatory evaluations that did not establish a causal association (29). In the present study, improvements in mood were independently associated with higher treatment satisfaction. However, this association should be interpreted cautiously, as it may reflect a bidirectional relationship. Similarly, the association between physical activity and satisfaction may reflect reciprocal effects rather than a direct causal pathway.

Furthermore, the significant association between positive social reactions and treatment satisfaction highlights the potential relevance of the social context in weight management. This finding is consistent with previous research highlighting the importance of social support and confirmation in promoting adherence and satisfaction with weight loss interventions (30, 31). Social reinforcement may contribute to patients’ subjective perception of treatment success; however, this association did not remain statistically significant in the fully adjusted model. This suggests that its effect may be mediated through other domains such as mood or self-perception.

Beyond psychological and social aspects, our study also captured appearance-related experiences. Some participants reported facial changes, including volume loss in the cheeks and temples, consistent with commentary on “Ozempic Face” after rapid GLP-1 receptor agonist-associated weight loss (32). However, perceptions varied: some viewed these changes negatively, whilst others reported improved self-image and body satisfaction. Facial changes were not associated with overall treatment satisfaction in multivariable analyses. Thus, unlike prior anecdotal reports (33, 34), these changes did not appear to be a key determinant of treatment satisfaction in this sample.

Although our findings offer valuable insights into patients’ experiences of incretin-based therapies, several limitations must be taken into account. First, the cross-sectional design precludes any conclusions regarding causality or temporal sequence. Second, as all data were self-reported, there is a risk of recall and social desirability bias. Furthermore, the anonymous online survey design limits the verification of clinical parameters, such as baseline and follow-up BMI, medication dosage, treatment duration, adverse events and self-reported comorbidities. Although rigorous data quality checks were implemented, self-reported measures are an accepted standard in patient-reported outcome research, particularly for subjective constructs such as treatment satisfaction and well-being. Third, recruitment via online platforms may have introduced selection bias, potentially favouring individuals with higher digital literacy and greater engagement with their therapy. This could restrict the applicability of the results to broader clinical populations, including older patients, those who are less digitally engaged, and those who are less active in online support communities. In addition, the relatively small size of the tirzepatide group may have limited the statistical power of the subgroup analyses. Furthermore, categorical variables were treated as continuous in the regression models, which implies an assumption of equal intervals between response categories and may introduce measurement bias. Finally, as the sample primarily comprised semaglutide and tirzepatide users, the results may not be generalisable to other GLP-1 receptor agonists or newer formulations, such as Zepbound.

## Conclusion

This study highlights the complex and multifaceted nature of treatment satisfaction amongst individuals using incretin-based therapies for weight management. Our findings suggest that treatment satisfaction is associated with tolerability, satiety, mood, and physical activity, but not with BMI reduction after adjusting for patient-reported treatment experiences. These results support the need for a broader evaluation of obesity pharmacotherapy that considers patient-reported outcomes beyond weight loss alone. From a clinical perspective, a comprehensive approach to treatment is needed to minimise treatment burden, promote psychosocial well-being, and encourage positive behavioural changes. Further longitudinal studies are required to clarify causal relationships and establish whether targeted interventions could enhance treatment satisfaction and treatment continuation.

## Data Availability

The raw data supporting the conclusions of this article will be made available by the authors, without undue reservation.
